# Vestibular therapy to reduce falls in people with Alzheimer’s disease: study protocol for a pilot randomized controlled trial

**DOI:** 10.1186/s40814-022-01133-w

**Published:** 2022-08-02

**Authors:** Lekha V. Yesantharao, Paul Rosenberg, Esther Oh, Jeannie Leoutsakos, Cynthia A. Munro, Yuri Agrawal

**Affiliations:** 1 Department of Otolaryngology-Head and Neck Surgery, Johns Hopkins University School of Medicine, Baltimore, MD 21205 USA; 2 Department of Psychiatry and Behavioral Sciences, Johns Hopkins University School of Medicine, Baltimore, MD 21205 USA; 3 Department of Medicine, Johns Hopkins University School of Medicine, Baltimore, MD 21205 USA

**Keywords:** Alzheimer’s disease, Fall-related injury, Vestibular therapy, Vestibular rehabilitation

## Abstract

**Background:**

Falls are highly common in patients with Alzheimer’s disease (AD); around two-thirds of AD patients fall annually. Fall events are major drivers of injury, early institutionalization, and shorter survival. Balance and mobility impairment are among the most important fall risk factors in AD patients. Vestibular therapy (VT) is an effective rehabilitation intervention in improving balance and fall risk through vestibular function, but not often used in AD. We want to evaluate the feasibility of using VT to reduce falls and improve balance function in patients with AD and drive use of an existing, potentially beneficial therapy in a patient population whose high level of vestibular deficits is currently unaddressed.

**Methods:**

The proposed pilot clinical trial will be a parallel-group randomized controlled trial. Patients with a diagnosis of mild-moderate AD, age ≥ 60, and the presence of a caregiver will be recruited from the Johns Hopkins Memory and Alzheimer’s Treatment Center. Eligible patients will be offered vestibular testing. Patients with vestibular loss will be offered participation in the VT trial. One-hundred AD patients with vestibular loss will be enrolled and randomized 1:1 into the control and intervention arms of the trial. All patients will undergo baseline balance and cognitive assessment, followed by 8 weeks of active control therapy or VT, consisting of ~25-min office sessions with a vestibular therapist. Patients will be tracked for falls and undergo follow-up balance and cognitive assessment at 8 and 52 weeks (1 year) to assess the potential short-term and longer-term effects, respectively, of VT on balance and cognition. The main outcomes of this trial are falls, balance (using the Berg Balance Scale and the Timed Up and Go test), and cognition (using the clock drawing test, the Card Rotations test, the Money Road Map test, and the triangle completion task).

**Discussion:**

As the population ages and the number of individuals with AD in the US grows to a projected 14 million in 2050, managing falls in AD will continue to grow as a critical public health concern; this trial assesses feasibility of a potential solution.

**Trial registration:**

ClinicalTrial.Gov identifier — NCT03799991. Registered 01 August 2019.

## Background

Falls are highly common in patients with Alzheimer’s disease (AD). Around two-thirds of patients with AD fall each year, which is double the annual fall rate among cognitively unimpaired older adults [1–3]. Falls are a major concern for AD patients and their families, given that they are major drivers of injury, early institutionalization, and shorter survival [4–9]. As the population ages and the number of individuals with AD in the USA grows to a projected 14 million in 2050 [10], managing falls in AD will continue to grow as a critical public health concern. Balance and mobility impairment are among the most important fall risk factors in AD patients, independent of dementia severity [1, 7, 11–13]. Emerging evidence demonstrates that significant balance and gait impairment is present even in the earliest stages of AD [14, 15]. Patients with mild-moderate AD have been shown to have greater postural instability on an unstable support surface or with a reduced base of support, as well as slower gait speed and higher gait variability, compared to age-matched controls [2, 16–18]. There is growing awareness that motoric impairment (in addition to cognitive impairment) may be a manifestation of the neurodegeneration associated with the development of AD [19–23]. The vestibular (inner ear balance) sensory system is known to play a critical role in the control of posture and gait and falls risk in cognitively normal adults [24–27]. The vestibular system consists of three semicircular canals (superior, posterior, and horizontal) which detect head movement and two otolith organs (saccule and utricle) which sense the orientation of the head with respect to gravity. The vestibular system is involved in two reflex pathways that are critical to maintaining balance: the vestibulo-ocular reflex (VOR) and the vestibulospinal reflex (VSR) [28–30]. Via the VOR, the vestibular system senses head movement and drives compensatory eye movements to keep the visual world stable during physical motion (e.g., walking or running). Via the VSR, the vestibular system senses the movement and orientation of the head and drives the trunk and lower extremity muscles to make compensatory postural adjustments to maintain upright balance. The symptoms of vestibular loss reflect impaired functioning of the VOR and the VSR and include unstable vision (oscillopsia) and impaired balance respectively [31, 32]. Of note, we define “vestibular loss” as a reduction and not total loss of vestibular sensory function, akin to vision or hearing loss. Vestibular loss is known to occur with healthy aging [26, 31–33]. This loss appears to be much more substantial in patients with AD, with a ~50% prevalence of vestibular loss in AD patients vs. ~25% in healthy older adults, for reasons that are not clear [34]. Additionally, vestibular loss in AD patients is largely going untreated.

Vestibular therapy (VT) is the mainstay of treatment for vestibular loss, supported by substantial evidence [35]. A recent Cochrane review concluded that there is moderate to strong evidence from high-quality randomized controlled trials that VT improves objective measures of balance (e.g., posturography), improves daily functioning, and reduces symptoms in individuals with vestibular loss [36]. In fact, a number of studies have shown that VT reduces the rate of falls ~20-40% [37–41], in both younger and older adults [42–44]. VT consists of a series of exercises designed to foster compensation for vestibular loss. Vestibular compensation occurs via 3 primary mechanisms: adaptation, substitution, and habituation [45]. With adaptation to vestibular loss, the central nervous system increases its responses to residual intact peripheral vestibular sensors [46]. With substitution for vestibular loss, other mechanisms (e.g., neck muscle proprioceptors) are recruited to replace vestibular contributions to the VOR and VSR [47–49]. With habituation, repeated exposure to provocative stimuli (e.g., fast movement such as running) over time results in a reduction of undesirable symptoms such as dizziness and imbalance [50]. VT is thus known to hasten and strengthen the mechanisms of adaptation, substitution, and habituation in order to improve balance and reduce fall risk in individuals with vestibular loss. Vestibular impairment is also associated with poorer spatial cognitive skills and potentially contributes to a “spatial” subtype of Alzheimer’s disease, characterized by wandering and falls [51]. Thus, VT may also help prevent or mitigate spatial cognitive decline as well. In fact, vestibular dysfunction is also correlated to cognitive impairments in nonspatial cognitive domains including problems in concentration, short-term memory, reading abilities, or executive functions (although it is not clear how VT targets spatial cognition versus other domains of cognitive function) [52]. Despite its potential benefits, VT is typically not offered to AD patients; in surveying our own clinical data, of the 1907 patients seen in the Johns Hopkins Memory and Alzheimer’s Treatment Center in 2018, < 1% (only 5 patients) were referred for vestibular therapy. This may be the case for a number of reasons, including the following: (1) infrequent assessment and diagnosis of vestibular impairment in AD clinical practice and (2) unclear benefit of VT in AD patients (although the benefit of VT on balance function and falls among cognitively intact older adults with vestibular impairment is well-established). As noted previously, vestibular loss in patients with AD is more common and more severe than in healthy older adults [34], and thus, the potential benefit of VT in AD patients may be even greater. Prior studies have shown that even patients with fairly severe AD are capable of motor learning and can improve their motor function following physical therapy [53–57]. Thus, we propose to systematically investigate whether VT can reduce falls and improve balance and spatial cognitive function in patients with AD. Our broader objective is to develop the evidence base needed to increase awareness of vestibular impairment in AD patients and drive use of an existing, potentially beneficial therapy in a patient population whose high level of vestibular deficits is currently going unaddressed.

The primary aim of this feasibility trial is to evaluate the trial design with regard to aspects such as method of recruitment, attrition rate, and assessing choice of primary outcomes for the main trial. This will be accomplished through the study of three secondary aims. The first is regarding falls. We will evaluate the feasibility of using VT to reduce falls in patients with AD. We hypothesize that VT will have preliminary efficacy in reducing 1-year incident fall rates relative to an active control intervention in a convenience sample of 100 patients with mild-moderate AD. The second is regarding balance. We will evaluate the feasibility of using VT in improving balance in patients with AD. We hypothesize that VT will have preliminary efficacy in improving balance as measured by the Berg Balance Scale (BBS) and the Timed Up and Go (TUG), relative to the active control intervention, at 8 weeks and 1-year after randomization. The third is regarding spatial cognition. We will evaluate the feasibility of using VT in improving spatial cognition in patients with AD. We hypothesize that VT will have preliminary efficacy in improving spatial cognition as measured by the Money Road Map, the Card Rotations, and the clock drawing tests, relative to the active control at 8 weeks and 1 year.

## Methods/design

### Design/setting

The overall research design of the proposed pilot clinical trial will be a single-blind parallel group randomized controlled trial to evaluate the feasibility of using VT in patients with mild-moderate AD. Patients will be recruited from the Johns Hopkins Memory and Alzheimer Treatment Center (JHMATC) in Baltimore, Maryland. The JHMATC is a clinical center collaboratively run by the departments of psychiatry, neurology, and geriatric medicine, based in the Johns Hopkins Bayview Medical Center. The JHMATC sees patients with a range of conditions that affect cognition and memory, including AD. Approximately, 700 new AD patients and 1800 follow-up AD patients are seen each year in the JHMATC. Patients are offered a comprehensive evaluation at each visit, access to innovative treatment through participation in research studies, and can sign HIPAA waivers allowing researchers access to their medical records for the purpose of study recruitment. AD diagnoses are made based on the National Institute on Aging–Alzheimer’s Association (NIA-AA) 2011 criteria. Patients with AD are categorized as having mild, moderate, or severe dementia based on their Clinical Dementia Rating (CDR) scores.

The inclusion criteria for this study are as follows: (1) diagnosis of AD based on the NIA-AA 2011 criteria that is mild-moderate (*CDR* = 0.5–2); (2) age ≥ 60 years; (3) vestibular loss defined as bilaterally impaired vestibular responses (abnormal semicircular canal or otolith responses bilaterally, defined below); (4) able to participate in study procedures including vestibular physiologic testing, balance and gait assessment, neurocognitive testing, and VT or active control; (5) sufficient cognitive capacity to give informed consent. We anticipate that individuals who are too impaired to provide informed consent would also not be able to effectively participate in VT or active control; and (6) the presence of a caregiver, defined as an individual who spends at least 10 h per week with the patient. Both the VT and active control involve 8 weeks of once weekly visits, and we believe a caregiver would increase the likelihood of successful completion of either therapy. The physical therapy intervention will be delivered by licensed and trained physical therapists who have experience working with patients with cognitive impairment.

Exclusion criteria for this study are as follows: (1) diagnosis of severe AD (*CDR* ≥ 3) [58]; (2) diagnosis of mild cognitive impairment or diagnosis of non-AD dementia, for example, Parkinson’s disease dementia, dementia with Lewy bodies, vascular dementia, frontotemporal dementia, and primary progressive aphasia; (3) deemed unable to participate in study procedures and VT or active control (e.g., patients with significant medical comorbidities, excessive agitation, or use of mobility aids such as a cane or walker); (4) use of daily vestibular suppressant medications, specifically antihistamines and benzodiazepines, as this can alter the response to VT [59, 60]; and (5) lack of availability to participate in 8 weeks of VT or active control.

### Recruitment

All eligible patients seen in the JHMATC (i.e., those with mild-moderate AD, age ≥ 60 years, presence of caregiver) will be offered vestibular physiologic testing. Patients may be new or follow-up patients in the JHMATC. We will comprehensively assess the function of the entire peripheral vestibular system, including the semicircular canals and otolith organs (saccule and utricle). In prior studies, we observed that rates of both semicircular canal and otolith function impairment are higher in AD patients relative to age-matched controls [34, 61, 62]. We have previously published details of vestibular testing, including in patients with AD [31, 32, 63–65].

In brief, semicircular canal function will be assessed using video-oculography, which records eye and head movement velocities during head impulses delivered by the examiner [65, 66]. An interacoustics 3D video-oculography system (Eden Prairie, MN) is used for vHIT testing. Semicircular canal function is measured as the ratio of eye velocity to head velocity, a quantity that is referred to as VOR gain. VOR gain values < 0.8 are considered abnormal. Saccular function will be assessed using the cervical vestibular-evoked myogenic potential (cVEMP). VEMP testing is performed with a portable multipurpose-evoked potential system (Medelec Synergy EMG/EP system, Viasys Health Care Systems, Old Woking, UK). TDH-49 calibrated headphones are used for sound-evoked cervical VEMP testing, and a triggered reflex hammer is used for vibration-evoked ocular VEMP testing. An acoustic tone burst is delivered to each ear, which stimulates the saccule. Response amplitudes (in μV) from the ipsilateral sternocleidomastoid myogenic response are recorded. Utricular function will be measured using the ocular vestibular-evoked myogenic potential (oVEMP). A forehead vibration stimulus is delivered which stimulates the right and left utricles simultaneously. Response amplitudes (in μV) from the contralateral inferior oblique myogenic responses are recorded (also simultaneously), given that the oVEMP is a crossed response. Absent cVEMP and oVEMP responses in either ear are considered impaired saccular and utricular function, respectively. We have successfully performed all three vestibular tests in over 100 patients seen in the JHMATC [34], and we published a technical paper with our observation that AD patients with a Mini-Mental State Exam (MMSE) score ≥ 13 could reliably comply with all vestibular testing procedures [63].

We anticipate based on our prior findings that 50% of eligible patients will have vestibular loss; these patients will be offered participation in the VT trial. Informed consent will be obtained from eligible participants at the time of the JHMATC clinic visit or subsequently over the telephone pending patient/caregiver preference and time availability. A study clinician will assess the patient’s capacity to make informed decisions about participating in this study and will complete a competency/capacity evaluation form. A member of the study staff will go through the informed consent form with the participant and caregiver and answer any questions regarding study-related procedures. Potential participants who decide to participate in the study will sign and date the consent. IRB approval for this study and the associated consent form have been obtained from the Johns Hopkins IRB. We will enroll 100 AD patients with vestibular loss who will be randomized 1:1 into the control and intervention arms of the trial. This is a superiority trial. The physical therapist delivering the intervention will know the treatment assignment; otherwise, all investigators, study staff, and outcome raters and assessors will be blinded to treatment assignment.

We will use a number of strategies to encourage adherence in our study. One strategy that has worked previously is providing free transportation. Also, we believe necessitating a caregiver will increase the likelihood of completion of either VT or active control therapy. Additionally, a study team member will contact patients weekly during the 8-week intervention to thank them for their participation in the study, remind them of their upcoming visit, inquire about any adverse events, and answer any questions.

### Staff training and data quality/storage

Trained study staff will perform the balance and cognitive outcome assessments. As per procedures in our research group, during the training period which takes 2–3 months, study staff are given increasing responsibility and independence in learning the assessment protocols. When study staff are deemed able to perform independently, typically duplicate measurements are performed in ~5 participants by study staff members and their trainers (e.g., the PI, co-investigators, and physical therapists) to assure inter-rater reliability on assessment procedures. All data collection protocols, and forms, are maintained on the study’s shared drive with the version number and date clearly indicated.

Specific data will be collected for this project including clinical data and results of balance and cognitive testing. Data will be collected by the research assistants and investigators by assessing and interviewing the subjects. All data will be entered into a custom REDCap data entry system and stored on a password-protected HIPAA compliant shared server. Access to identifiable private information will be restricted to study personnel, including study investigators and research staff. Quality control during data entry will be assured by the following: (1) double data entry of key fields, e.g., balance function scores, (2) automatic prompts to resolve discrepancies in double-entered values, (3) ensuring data entries are within allowable ranges, and (4) database-generated reports of all values missing in the form. All data will be automatically backed up on the Hopkins server daily. Monitoring reports to gauge data quality will be generated and reviewed monthly. Monthly reports will be generated to track completion of all scheduled visits; any missed visit will be reported to the study coordinator who will follow-up to ascertain the reason for the missed visit and reschedule the visit if possible. A detailed codebook will be generated in REDCap delineating question by question how responses are coded in the database and allowable entries and ranges for each field.

### Interventions

We chose an active comparator/control regimen that consists of stretching and toning (ST) exercises (e.g., range of motion exercises, lifting light weights with the arms and legs) that rotate weekly. The use of an active control group will minimize potential practice effects on the outcome assessments and also minimize any expectancy effects on the part of the participant. This ST regimen is “vestibular neutral” in that head movements which specifically challenge the vestibular system are avoided. This regimen is frequently used as an active control in AD intervention trials and will be delivered by the physical therapist at the same dose (i.e., weekly office visits over 8 weeks) as VT [67]. Additionally, at first visits with both the control and treatment groups, auditory function will be measured using mobile SHOEBOX Audiometry, a clinically validated tablet audiometer [68]. This will allow us to account for differences in hearing levels between the two groups in later analyses.

The intervention itself will consist of 8 weekly 25-min sessions with a vestibular physical therapist. The recommended course of VT in individuals with chronic vestibular loss ranges from 8–12 weeks of therapy delivered in weekly office visits [69]. These same recommendations are also made for older adults [42]. In this study, we are choosing 8 weeks of therapy with once-weekly visits by the study staff to balance efficacy with real-world effectiveness for AD patients and their caregivers. As per typical physical therapy-based interventions, VT programs will be customized for the patient, and a therapeutic plan will be developed involving the core elements of vestibular physical therapy, including gaze stabilization exercises, balance and gait exercises, and/or habituation exercises (as established in the American Physical Therapy Association neurology section’s clinical practice guidelines regarding treatment content) based on the therapist’s assessment of the patient’s needs [69]. Exercises will be advanced along a standard regimen according to the patient’s progress. The standard regimen of weekly exercises will be as follows:

- Week 1: ×1 horizontal head movement (patients, while seated, will start a metronome at a pace of 60 beats per minute and hold a photo of their favorite pet or family member at arm length. They will keep eyes on the photo and move their head back and forth in the yaw-wise direction for 2 min, trying to complete a full head turn within each beat of the metronome); ×1 vertical head movement (the same activity as the one previously described, except the head movements are in the pitch-wise direction); standing with feet a comfortable distance apart with eyes open for 2 min; walk with head turns (for 2 min, the patient will try to walk at a consistent speed and in a straight line down a hall while turning their head from side to side in the yaw-wise direction); and walk with head tilt (the same activity as the one previously described, except the head movements are in the pitch-wise direction).
- Week 2: ×2 horizontal head movement; ×2 vertical head movement; standing with feet together with eyes open for 2 min; walk with head turns, focusing on keeping along a narrow, straight path; and walk with head tilt, focusing on keeping along a narrow, straight path.
- Week 3: Horizontal gaze shifting exercise (patients will start their metronome at a pace of 60 beats per minute and hold two photos at arm’s length about 8 inch apart. After aligning their head and eyes with the first photo, they will turn their eyes only and then their head to look at the second photo. Then they will turn their eyes first, followed by their head, and back to the first photo, attempting to complete one gaze shift within each metronome beat. This gaze shifting in the yaw-wise direction will be continued for 2 min); vertical gaze shifting exercise (the same activity as the one previously described, except the head movements are in the pitch-wise direction); standing with feet in semi-tandem position and eyes open for 2 min; and walk with head turns (in a hallway, the patient will fix their eyes on a target on a wall directly ahead of them and try to walk at a consistent speed and in a straight line down the hall while turning their head from side to side in the yaw-wise direction, keeping their eyes fixed on the target at all times. After 5 steps forward, the patients will try to walk 5 steps backwards while maintaining the same head movements. This will be repeated for 2 min).
- Week 4: Imaginary target exercise standing with feet in semi-tandem (patients will stand with their feet together but staggered, looking at a photo held directly in front of them. They will close their eyes and turn their head slightly in the yaw-wise direction, imagining that they are still looking directly at the photo. They will open their eyes and check to see if they are still successfully looking at the photo. This will be repeated in the opposite direction and continued for 2 min. This whole exercise will then be repeated in the pitch-wise direction, standing with feet in tandem with eyes open for 2 min, and walk in a “figure-8” pattern (patients will place 2 small chairs about 6 feet apart and walk in between and around each chair to create a “figure-8” pattern for 2 min).
- Week 5: Same activities as week 1 but standing on foam cushion for all tasks except for the walking tasks.
- Week 6: Same activities as week 2 but standing on a foam cushion for all tasks except for the walking tasks.
- Week 7: Same activities as week 3 but standing on a foam cushion for all tasks except for the walking task.
- Week 8: Same activities as week 4 but standing on a foam cushion for all tasks except for the “figure-8” walking task.

Each weekly set of 4 exercises will be performed 3 times on the day of VT (2 min per task, 8 min per set, ~24 min total for 3 repetitions of the set of exercises). Further detail on vestibular therapy exercises is provided in Reference 35, with explanations for adjustments made to standard VT for individuals with cognitive impairment in Reference 70 [35, 70]. Cueing of patients as needed to assist with task completion will be performed, following the VT in AD protocol we recently published [71]. In our own pilot data, we observed an improvement in spatial cognitive skills in 5 AD patients who underwent vestibular therapy, emphasizing the feasibility of this protocol. No participant will be removed from the study unless he or she wishes to terminate his or her participation. Any participant can end participation at any time.

All participants will receive routine care and will be offered this new intervention on a voluntary basis. Currently, vestibular loss is largely undetected and untreated in patients with cognitive impairment and AD, so this is considered a new intervention additional to routine care.

#### Outcomes

**Table:** 

Assessments	Screening	Baseline	8 weeks	52 weeks
*Vestibular testing*				
Saccule	x		x	x
Utricle	x		x	x
Semicircular canals	x		x	x
*Falls*		x	x	x
*Fall risk factors*				
Medications		x	x	x
Strength		x	x	x
Other sensory (vision, proprioception)		x	x	x
*Balance testing*				
Berg Balance Scale		x	x	x
Timed Up and Go		x	x	x
*Cognitive testing — spatial*				
Clock drawing test		x	x	x
Card Rotations test		x	x	x
Money Road Map test		x	x	x
Triangle completion task		x	x	x
*Cognitive testing — other domains*				
RBANS		x	x	x
MMSE		x	x	x
Trail Making Test		x	x	x
COWAT		x	x	x

The endpoint for the current proposal is an incident fall, defined as a participant “coming to rest inadvertently on the ground or other lower level” [72]. We will ascertain fall events using monthly calendars, which are considered the gold standard of prospective fall measurement. Patients and caregivers will be given 12 self-addressed stamped envelopes to return each monthly calendar. If we do not receive a calendar within 2 weeks of the expected receipt date, we will call the patient by telephone. For any fall that is reported, we will collect standardized information including any injuries that occurred, per the procedures developed for the STRIDE trial [73]. Additionally, given that multiple risk factors have been shown to contribute to falls in AD patients, we will also measure other known fall risk factors, including history of falls, medications (number of CNS-active medications and anticholinergic burden), strength (using hand dynamometer), and other sensory factors (vision assessed using Snellen visual acuity and Pelli Contrast Sensitivity charts; proprioception assessed using Romberg test), based on published procedures in AD patients [74–80].

In order to measure outcomes related to balance, we have chosen the Berg Balance Scale (BBS) and the Timed Up and Go (TUG) test because both tests have been well-validated in AD patients, are easy to administer (~5–10 min for both tests), and have shown excellent test-retest reliability among patients with mild-moderate AD (intra-class correlation coefficient for both the BBS and TUG ≥ 0.9) [81–85]. Studies showed that patients with mild-moderate AD were able to complete all of the balance tests, although some participants required additional cueing to remember steps in the test instructions. The BBS assesses the ability to maintain balance during a set of 14 simple, everyday tasks. Each item is graded on a 5-point scale, ranging from zero (patient unable to perform task) to four (patient can independently complete the task for the full duration), for a total possible score of 56. Additionally, given the increased sensitivity of quantitative postural testing in detecting mild balance impairment in AD patients (18), we will measure center-of-mass sway during standing tasks in the BBS using the BalanSens™ posturography system per established procedures in our laboratory [86]. The TUG test is a widely used clinical test that incorporates a series of tasks critical for independent mobility: standing up from a seated position, walking 3 meters, turning back around, walking, stopping, and sitting down [87]. Subjects are instructed to perform the task as quickly as is safely possible, and the time taken from bottom off the seat to bottom back on the seat is measured using a stopwatch. In this study, the mean time to completion of two trials of the TUG will be recorded.

Measures to assess cognitive outcomes will include assessments of spatial and nonspatial cognitive function. We selected the clock drawing test, the Card Rotations test, the Money Road Map test, and the triangle-completion task as spatial cognitive measures that assess various aspects of spatial cognition (including mental rotation, spatial memory, and spatial navigation). These tests can be given to patients with a wide range of cognitive deficits and altogether take ~10–15 min to administer. The clock drawing test requires patients to draw an analog clock and draw the hands to depict a specified time [88]. The clock drawing test assesses conceptually guided constructional praxis and also requires executive skills. Drawings are scored based on accuracy, with consideration of spacing of the numerals within the clock face, completeness and correctness of numerals, and hand placement. In the Card Rotations test, subjects are shown a reference shape, followed by a series of similar objects that are variously rotated [89]. Subjects are asked to mentally rotate the objects to determine whether they are identical to or mirror images of the reference shape. The score is the number of correct responses minus the number of incorrect responses completed within 3 min. In our prior work, we found that performance on this test was strongly correlated with vestibular function in healthy older adults [61]. The Money Road Map test involves a map with a path that has 32 corners. Patients are asked to imagine themselves walking along a path and indicate when they get to a corner if they are to turn right or left to stay on the path [90]. This test assesses the ability to make egocentric mental rotations (i.e., in reference to one’s own point of view). The test is scored based on the number of errors made (range 0–32). Patients with AD have been shown to perform worse on this test compared to control subjects, and the range of performance on this test has been shown to vary widely among AD patients [91]. Moreover, we recently reported that AD patients with vestibular loss had significantly poorer performance on this test relative to AD patients with intact vestibular function [92]. The triangle-completion task (TCT) measures dynamic spatial navigation. Patients are moved along two limbs of a triangle wearing a blindfold and are asked to turn and return to the perceived origin of the triangle on their own. This test has been extensively used in older adults (including in the Baltimore Longitudinal Study of Aging) as well as in patients with vestibular disorders [93–95]. We will also explore the effect of VT vs. active control on nonspatial cognitive tests, measured with the Repeatable Battery for Neuropsychological Status (RBANS), focusing on the immediate and delayed memory, language, and attention domains [96]. We will also administer the Trail Making Test (TMT) and the Controlled Oral Word Association Test (COWAT) as measures of executive function and the MMSE as a global measure of cognitive function [97–99].

We propose to evaluate fall events over a 1-year follow-up period given that prior studies demonstrating feasibility of using VT have shown benefit in reducing fall rates and improving balance outcomes up to 12 months following VT [36]. Of note, we will be collecting monthly falls data, such that if there is an initial difference in fall rates between the 2 groups that starts to narrow during the follow-up period, we can consider including “booster” VT sessions in subsequent trials. Additionally, we will evaluate short-term balance and cognitive outcomes at the completion of the 8-week course of VT. Prior studies of VT in patients with chronic vestibular loss have shown improvements in balance and gait performance when assessed from 8–16 weeks after baseline following an 8-week course of VT [100–102]. In order to explore the longer-term “disease-modifying” effects of VT in AD patients, we will also secondarily assess balance and cognitive outcomes at 1 year. Furthermore, we will consider whether balance and cognitive function during the follow-up period mediate the association between VT and 1-year incidence of falls. We will also assess vestibular function at 8 weeks (short term) and 52 weeks (long term) given that further deterioration of the vestibular apparatus may influence VT outcomes.

### Sample size

As mentioned previously, we will enroll 100 AD patients with vestibular loss who will be randomized 1:1 into the control and intervention arms of the trial.

We note that the goal of this pilot trial is to determine design feasibility and preliminary efficacy, i.e., estimate effect size, of VT on 1-year falls incidence. As such, we are choosing a convenience sample of 100 patients, to balance the competing considerations of enrolling a sufficient sample size to overcome heterogeneity in fall rates, though not committing undue effort and resources to an intervention that still requires higher level of evidence before conducting a definitive phase 3 trial. We used the recurrent event data simulation algorithm developed by Jahn-Eimermacher et al. [103] to estimate the power associated with enrolling a convenience sample of 100 participants. We made the following assumptions based on the literature and prior experience: (1) 70% of untreated individuals will experience at least 1 fall during the year of follow-up; (2) those who fall experience an average of 3 falls per year [104]; (3) the rate for loss-to-follow-up over 2 years is 20% (a conservative estimate based on prior studies in the JHMATC, with losses to follow-up due to patient relocation, institutionalization, and death), and the dropout follows a uniform distribution over the 2-year interval. The effect sizes (hazard ratios) of 0.4, 0.5, and 0.6 (associated with enrolling 100 participants) correlate with powers of 95%, 80%, and 56%, respectively. As a frame of reference, in cognitively intact older adults, multifactorial fall risk reduction interventions have reduced fall rates by ~40–50% [105, 106], and single interventions such as cataract surgery have reduced fall risk by 30–40% [107]. Additionally, we tested the effect on our statistical power of adding the other baseline covariates to adjusted models. Compared to the crude analysis, we will gain power by including confounders that have a meaningful association with falls (e.g., assuming that the combined confounders increase the odds of falls by ~threefold). We will balance the study arms on age and baseline MMSE using dynamic minimization software developed by the study statistician [108].

### Statistical methods

With regard to the primary objective of determining the feasibility of this study design, descriptive statistics on the patient population recruited will be summarized to understand how effective our recruitment and attrition strategies have been. Continuous variables (such as age) will be summarized as means and associated standard deviations if normally distributed or medians and associated ranges for skewed data. Categorical data will be summarized as counts and percentages.

With regard to the falls-related data, we will treat incident fall events as recurrent events such that the sequence of times of the recurrent event (i.e., repeated falls) constitutes a simple point process recorded continuously over the 1-year follow-up period. We will model the intensity of the recurrent events using the Andersen-Gill (AG) model [109], which generalizes the Cox proportional hazard model for counting processes. This model will also be adjusted for baseline covariates including age, sex, hearing, disease duration, fall risk factors, balance, and global cognition. In secondary analyses, we will include interaction terms between these covariates and treatment assignment to assess possible predictors of treatment response. For example, the coefficient for the interaction between female sex and VT would be the difference in treatment effect between females and males.

With regard to balance, to evaluate whether participants improve after therapy, we will fit a longitudinal random effects model with a random intercept such that E[*y*_ij_] = *β*_0_ + *β*1 visit + *β*_2_ VT + *β*_3_ VT × visit, where *y*_ij_ is the outcome (BBS or TUG) for the *i*th participant at the *j*th visit (either baseline (0) or post treatment (1)), *β*_0_ is the fitted mean outcome at baseline for individuals in the active control arm, *β*_1_ is the fitted change in outcome comparing post- to pre-treatment visits among those in the active control arm, *β*_2_ is the between-arm difference at baseline (assumed to be 0), *β*_3_ is the between-arm difference in change in outcome over time, and can be interpreted as the treatment effect. The regression model will also be adjusted for baseline covariates including age, sex, hearing, disease duration, balance, cognition, and fall risk factors. In secondary analyses, we will include interaction terms between these covariates and visit × VT to assess possible predictors of treatment response.

We will also explore whether changes in balance and/or cognition during the follow-up period account for (i.e., mediate) the impact of VT on falls. The models described in aim 2 will be similarly applied here to evaluate change in cognitive function following VT. If there is an effect VT on cognitive function, we will assess the relationship between VT and primary outcomes after adjusting change in cognition. Secondarily, we will fit similar models to the nonspatial cognitive outcomes. If there is appreciable improvement in nonspatial cognitive outcomes, we will regress nonspatial change scores on spatial change scores (adjusting for baseline scores) to determine if gains in nonspatial domains are correlated with spatial gains.

### Oversight and monitoring

The trial steering committee will consist of the study principal investigator and co-investigators. The steering committee will meet monthly and will be responsible for trial oversight, enrolment, participant safety, data management, outcome adjudication, and, once the trial is completed, reporting and dissemination of study findings. The steering committee will oversee the work of the physical therapist(s) delivering the intervention and research study staff. The steering committee will also maintain robust communication with the Data Safety and Monitoring Board (DSMB).


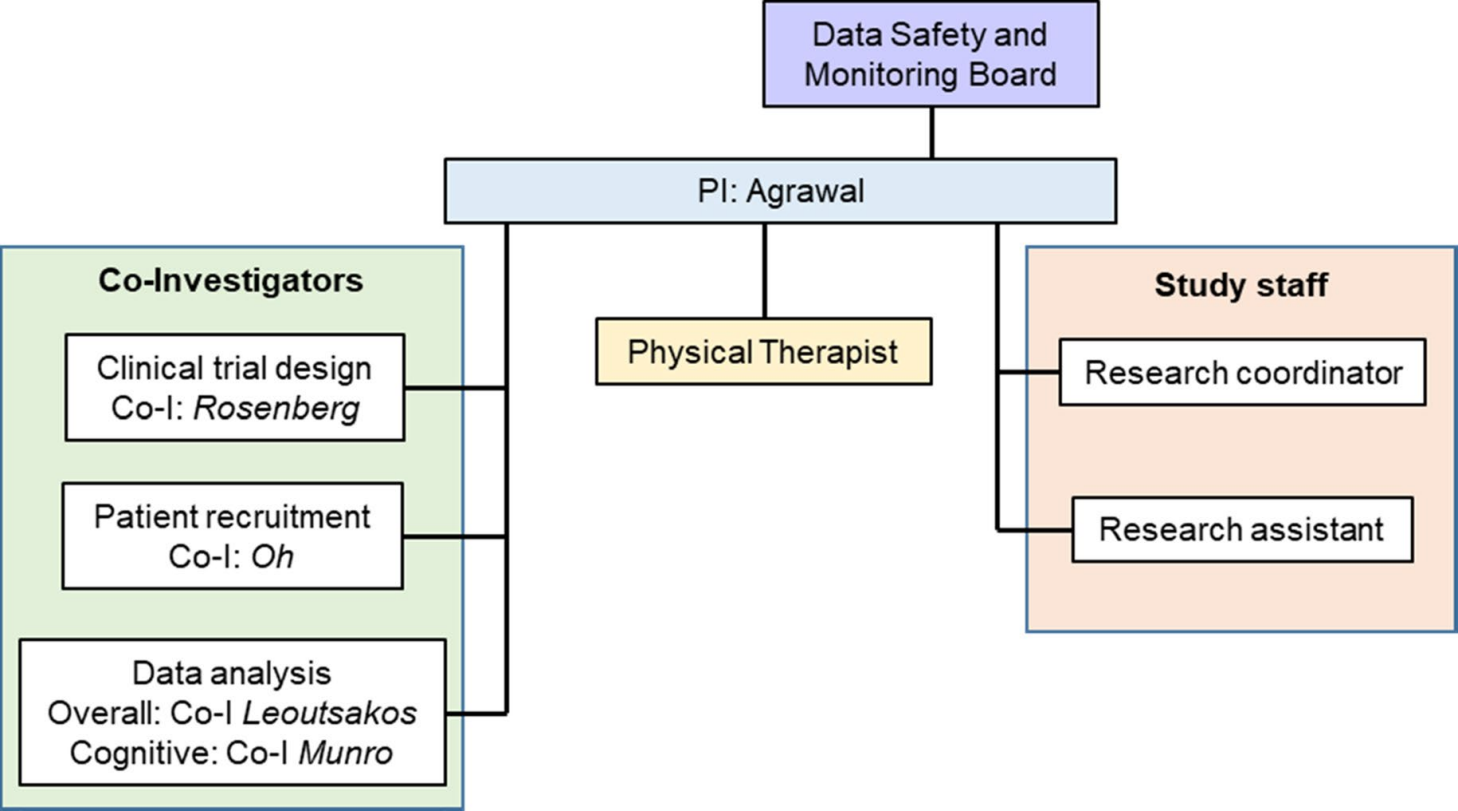


The DSMB will act in an advisory capacity to the NIA director to monitor participant safety, evaluate the progress of the study, to review procedures for maintaining the confidentiality of data, the quality of data collection, management, and analyses. The following individuals have accepted positions as part of the DSMB. DSMB membership has been reviewed and approved by the National Institute on Aging.

DSMB members will have no direct involvement with the study investigators or intervention. Each DSMB member has signed a conflict of interest statement which includes current affiliations, if any, with pharmaceutical and biotechnology companies (e.g., stockholder, consultant) and any other relationship that could be perceived as a conflict of interest related to the study and/or associated with commercial interests pertinent to study objectives. The DSMB will meet twice annually, either in person or by teleconference call to review study progress, data quality, and participant’s safety. The main responsibilities of the DSMB include the following:

- Reviewing the research protocol/informed consent documents/plans for data safety and monitoring
- Advising the NIA on the readiness of the study staff to initiate recruitment
- Evaluating the progress of the trial, including periodic assessments of data quality and timeliness, recruitment, accrual and retention, participant risk versus benefit, performance of the trial sites, and other factors that can affect study outcome
- Considering factors external to the study when relevant information becomes available, such as scientific or therapeutic developments that may have an impact on the safety of the participants or the ethics of the trial
- Reviewing study performance and making recommendations and assisting in the resolution of problems reported by the principal investigator
- Protecting the safety of the study participants
- Reporting to NIA on the safety and progress of the trial
- Making recommendations to the NIA and the principal investigator concerning continuation, termination, or other modifications of the trial based on the observed beneficial or adverse effects of the treatment under study
- Ensuring the confidentiality of the study data and the results of monitoring
- Assisting the NIA by commenting on any problems with study conduct, enrollment, sample size, and/or data collection

All clinical personnel, including the PI, co-investigators, and the physical therapist, will be responsible for the safety of study participants. An adverse event (AE) is any adverse change from the participant’s baseline condition, regardless of relationship to study participation, or abnormalities which occur after informed consent is signed and up to 30 days after the study has been completed. AEs in this study will include but are not limited to the following: (1) intercurrent illness; (2) participant deterioration due to primary illness (i.e., cognitive impairment); and (3) new symptom or worsening of an existing symptom (e.g., pain). Each AE is evaluated for duration, severity, seriousness, and causal relationship to study procedures. The study physical therapist will report any AEs (e.g., headache, leg pain, and neck discomfort) associated with the VT or active control that occurs during the patient visits. Study staff will also query about the occurrence of AEs and serious AEs (defined below) at the weekly telephone call with the patient. A serious adverse event (SAE) is any untoward medical occurrence that results in death, is life-threatening, requires inpatient hospitalization or prolongation of existing hospitalization, or results in persistent or significant disability/incapacity. SAEs in this study will include but are not limited to the following: (1) inpatient hospitalization, (2) change in ability to conduct daily life functions, and (3) death. Each SAE will be evaluated by the study team and DSMB for duration, severity, seriousness, and causal relationship to study procedures.

Records of adverse events will be compiled and sent to the IRB and DSMB every 6 months. Study staff will notify the investigators immediately of a SAE, and a provisional report will be sent to the IRB and DSMB as the details of the SAE are collected. A final report will be sent to the IRB and DSMB once more definitive information surrounding the cause of death has been obtained. The PI and study co-investigators (in conjunction with the DSMB as necessary) will evaluate every adverse event for safety and causality and will determine whether the adverse event affects the risk/benefit ratio of the study and whether modifications to the protocol or consent form are required. In the extreme event that knowing the patient’s treatment assignment is relevant for the patient’s care, the physical therapist will inform the investigators about the treatment assignment and report the unmasking event to the IRB and DSMB. The DSMB will meet and review adverse events and SAE’s every 6 months, and an ad hoc DSMB meeting may be convened if an SAE may possibly be related to participation in the study.

Audit of the trial will be conducted every 6 months by the Data Safety and Monitoring Board. Specific reporting forms have been provided by the study sponsor, the National Institute on Aging, which provides a template for the DSMB to conduct the audit. Specific elements that we will report and will be reviewed by the DSMB include the following: study status, enrollment, participant descriptive information, protocol deviations, safety information (including occurrence of adverse events and serious adverse events), and study quality. DSMB meetings will have both an open component (with an associated reporting form) during which study investigators can present study reports to DSMB members. Additionally, DSMB meetings will have a closed component (with an associated reporting form), whereby DSMB members can discuss the trial independently from the investigators and sponsors and relay any feedback to the investigators and/or sponsor.

## Discussion

This study’s innovative application of an existing intervention, vestibular therapy, to AD patients expands upon past research in two ways. First, we focus on the motoric impairments in AD, extending beyond the primary focus on cognitive deficits. A growing literature is documenting the motoric impairments that occur concomitantly with cognitive impairments during AD progression [22, 110, 111]. AD patients have been shown to have significantly higher levels of balance impairment, mobility disability, spatial disorientation, and falls relative to healthy older adults [22, 112, 113]. Injuries resulting from falls can be devastating to patients with AD [3]. Indeed, the excess mortality in AD patients has been attributed to their increased risk of falls [9]. Despite increasing recognition of the substantial motoric impairments associated with AD, balance and mobility assessments are not routinely performed as part of the ongoing clinical evaluation of AD patients, which typically focuses on the trajectory of cognitive decline. There is a tremendous opportunity to incorporate motoric measures, such as balance function and gait, and antecedents of these measures, such as vestibular function, into the standard evaluation of AD patients. This is particularly true if effective, low-risk interventions such as VT may improve the trajectory of these motoric outcomes.

A second innovative feature of this proposal is that we will evaluate whether VT improves not only balance function, which is the standard target of VT, but also spatial cognitive skills in patients with AD. Growing evidence suggests an important link between vestibular and cognitive function, specifically spatial cognitive skills, in healthy older adults and in AD patients [51, 95]. Several studies, including our own pilot data, have shown an improvement in spatial cognition following VT in adults with vestibular loss. We hypothesize that VT fosters improved perception of head orientation (e.g., via substitution mechanisms, whereby neck proprioceptors are increasingly recruited to detect head orientation), which in turn improves the accuracy of spatial encoding and navigation. Whether VT improves spatial cognitive function or even other domains of cognitive function (which we will consider) remains to be established. This question is particularly relevant for AD patients who have disproportionate impairments in spatial cognitive abilities.

## Data Availability

The final trial dataset will be accessible to study investigators and staff only for 1 year after study completion. Following that, the data will be available upon request.

## Abbreviations

AD: Alzheimer’s disease
VT: Vestibular therapy
VOR: Vestibulo-ocular reflex
VSR: Vestibulospinal reflex
BBS: Berg balance score
TUG: Timed Up and Go
JHMATC: Johns Hopkins Memory and Alzheimer Treatment Center
NIA-AA: National Institute on Aging–Alzheimer’s Association
CDR: Clinical Dementia Rating
cVEMP: Cervical vestibular-evoked myogenic potential
oVEMP: Ocular vestibular-evoked myogenic potential
PI: Principal investigator
MMSE: Mini-Mental State Exam
ST: Stretching and toning
PCORI: Patient-Centered Outcomes Research Institute
STRIDE: Strategies to Reduce Injuries and Develop confidence in Elders
TCT: Triangle completion task
RBANS: Repeatable Battery for Neuropsychological Status
COWAT: Controlled Oral Word Association Test
ADRC: Alzheimer’s Disease Research Center
AG: Andersen-Gill
SAEs: Serious adverse events
IRB: Institutional Review Board
DSMB: Data Safety and Monitoring Board
